# Factors associated with low bone density in patients referred for assessment of bone health

**DOI:** 10.1186/1687-9856-2013-4

**Published:** 2013-02-06

**Authors:** Lisa Swartz Topor, Patrice Melvin, Courtney Giancaterino, Catherine M Gordon

**Affiliations:** 1 Division of Endocrinology, Boston, MA, USA; 2 Program for Patient Safety and Quality, Boston, MA, USA; 3 Division of Adolescent Medicine, all at Boston Children’s Hospital, Boston, MA, USA; 4 Harvard Medical School, Boston, MA, USA; 5 Divisions of Adolescent Medicine and Endocrinology, Hasbro Children’s Hospital, Warren Alpert Medical School of Brown University, Providence, RI, USA; 6 Research Associate, Boston Children’s Hospital, Boston, MA, USA

**Keywords:** Bone health, Low bone mineral density, Chronic disease, Vitamin D, Dual X-ray absorptiometry

## Abstract

**Background:**

To identify factors that predict low bone mineral density (BMD) in pediatric patients referred for dual-energy x-ray absorptiometry assessments.

**Methods:**

This is a retrospective cohort study of 304 children and adolescents referred for dual-energy x-ray absorptiometry assessments at a tertiary care center. Outcomes included risk factors which predicted a significant low bone density for age, defined as BMD Z-score ≤ -2.0 SD. A univariate analysis involved Chi-square, Fisher’s Exact test, and analysis of variance, and multivariate logistic regression models were constructed to determine predictors of low bone mineral density.

**Results:**

In the multivariate logistic regression model, predictors of low bone mineral density included low body mass index Z-score (odds ratio 0.52, 95% confidence interval 0.39 – 0.69), low height Z-score (OR 0.71, 95% CI 0.57 – 0.88), vitamin D insufficiency (OR 3.97, 95% CI 2.08 – 7.59), and history of bone marrow transplant (OR 5.78, 95% CI 1.00 – 33.45).

**Conclusions:**

Underlying health problems and associated treatments can impair bone mineral accrual. We identified risk factors most predictive of low bone mineral density in subjects referred for bone density measurement. Recognition of these factors may allow for earlier assessment to maximize bone mass in at-risk children.

## Background

In 2010, the Boston Children’s Hospital Program for Patient Safety and Quality convened a Task Force to identify the frequency of fragility or insufficiency fractures in hospitalized infants, children and adolescents and to reduce the risk of these fractures. As low bone density has been identified as a risk factor for fractures in children [1,2] a component of this bone health safety initiative was to identify factors associated with low bone mineral density in patients seen in our institution.

Specific pediatric populations are known to be at high risk for a low bone density, including children and adolescents with cerebral palsy and other non-ambulatory states [1-4], chronic renal failure [5], malnutrition and malabsorptive states [6-8], cystic fibrosis [9-11], pubertal delay [12], and 25-hydroxyvitamin D (25OHD) deficiency [13,14]. In addition, medical therapies such as treatment with anticonvulsants [15], glucocorticoids [15], and chemotherapy [16,17] are associated with a compromise of bone density. As fracture risk in children is inversely related to bone density in some reports [18-22], understanding the risk factors associated with low bone mineral density may provide greater opportunities for early identification and intervention for those at risk for skeletal fragility. To our knowledge, no study has examined the relative risk of these conditions and treatments on low bone density in a pediatric population.

Dual-energy x-ray absorptiometry (DXA) is a common methodology used to quantify bone mineral density (BMD), and provides a measure of bone mineral (g) per projected area scanned (cm^2^). In children, a Z-score is used to compare a child’s BMD with an age- and gender-matched norm, with appropriate adjustments for bone age or pubertal status often needed [20]. Guidelines established by the International Society for Clinical Densitometry define a low bone density as a BMD Z-score of less than or equal to -2.0 SD [23].

The objectives of this study were to determine risk factors for low bone density (BMD Z-score ≤ -2) in a pediatric population, and to identify which risk factors have the strongest correlation with BMD. A secondary aim was to examine whether fracture history was correlated with BMD Z-scores in children and adolescents who are within designated risk groups for low bone density.

## Methods

### Subjects

We identified all patients between the ages of 4-21 years who were referred to the Bone Health Program at Children’s Hospital Boston for BMD measurements by DXA as part of routine clinical care from October 2008 to September 2009. Patients who had the DXA performed as part of a research study were excluded. Subjects were categorized based on their lowest BMD Z-score and divided into three groups: BMD Z-score > -1.0 SD, between -1 to -1.9 SD, or ≤ -2.0 SD. Based on the results of a power analysis (described in ‘Statistical Analysis’), we reviewed charts consecutively by scanning date until we identified 100 subjects in each of the three subgroups (532 charts reviewed). Once 100 subjects were identified in a group, additional subjects in that group were not included. The Boston Children’s Hospital Committee on Clinical Investigation approved this protocol.

### Densitometry measurements

Scans were performed on a single densitometer. Areal (two-dimensional) bone density was quantified by DXA using a Hologic Discovery A scanner [Hologic Inc, Bedford, MA]. Bone mineral density (BMD, g/cm^2^) measurements were obtained at the left total hip and lumbar spine (L1-L4), and in some cases, at the whole body. Pediatric normative data were used to calculate BMD Z-scores at each skeletal site [24] using pediatric software, to allow for comparison with age- and gender-matched controls [24]. With this instrument in our DXA Center, the average *in vivo* precision for aBMD (expressed as percent coefficient of variation) was 0.62% at the spine and 0.72% at the total hip in children and adolescents.

### Data collection

Height and weight were obtained using a calibrated stadiometer (Kalamazoo, MO) and scale. Body mass index (BMI) was expressed as body weight in kilograms divided by the square of height in meters (kg/m^2^) as a weight-for-height index and was converted to percentiles and corresponding Z-scores by using age- and gender-specific normative values for US children [25]. We used the normative values for maximal age (20 years) [25] to calculate BMI for older subjects. Underweight was defined as BMI < 5^th^ percentile and overweight was defined as BMI > 85^th^ percentile for age and gender. Demographic and medical history data and DXA reports were obtained through a retrospective chart review of the Boston Children’s Hospital medical record. Data were collected from outpatient clinic notes, radiology reports, and DXA reports. Data included ethnicity, gender, fracture history, age at menarche, history of 25OHD insufficiency (defined as 25OHD level < 30 ng/mL, the lower limit of the normal range for our clinical laboratory), family history of osteoporosis, and history of prematurity. We recorded 25OHD values for subjects with 25OHD insufficiency. Information about specific medical conditions and treatments was collected from the medical record.

Data recorded from each subject’s DXA report included the absolute BMD and corresponding Z-scores at the left total proximal hip and lumbar spine (L1-L4). Only selected patients had total body DXA measurements, as the DXA measurements for many patients within this sample were performed prior to published recommendations by pediatric experts regarding preferred skeletal sites [23]. When available, DXA measurements adjusted for bone age were recorded when bone age differed significantly from chronological age. Additional data collected from the DXA report included the date of the study, the subject’s age, height, and weight at time of the BMD measurements, the indication(s) for the scan as provided by the referring physician, and the total number of prior DXA scans the subject had undergone.

### Statistical analysis

A two proportion power analysis was used to determine the minimum number of cases and controls necessary to detect a 15 percentage point difference in risk factors associated with low BMD Z-score (power = 0.8, alpha = 0.05). We determined that a minimum of 100 cases (BMD Z-score ≤ -2) and 200 controls (BMD Z-score > -2) would give our study the power necessary to detect this difference. For the descriptive analysis, patients were stratified into three groups based on the patient’s lowest BMD Z-score (for multiple DXA readings): > -1.0 SD, between -1 to -1.9 SD, or ≤ -2.0 SD. Patient demographics among the BMD Z-score groups were summarized using means and standard deviations for continuous variables and proportions for categorical variables. Statistical differences across the three groups were analyzed using Pearson’s chi-square or Fisher’s Exact test for categorical variables and one way analysis of variance (ANOVA) for continuous variables. Additionally, we assessed differences across DXA indications by gender using Pearson’s chi-square or Fisher’s Exact test for categorical variables.

For the univariate analysis, we dichotomized the BMD Z-scores into two groups: ≤ -2 and > -2 and assessed individual factors that may be associated with low BMD Z-score. Pearson’s chi-square and Fisher’s Exact test were used for categorical variables and ANOVA was used for continuous variables. Variables in the univariate model with a p-value ≤ 0.05 were considered for inclusion in a multivariate logistic regression model. A gender specific sub-analysis looking at fracture history and low BMD was performed using the Pearson’s chi-square test and ANOVA to assess differences between BMD groups. All analyses were performed using SAS software version 9.2 (SAS Institute Inc, Cary, NC), and a 2-sided p value ≤ 0.05 was considered indicative of statistical significance.

## Results

We obtained information on 304 children and young adults between the ages of 4-21 years old who underwent DXA scans at Boston Children’s Hospital. Height and weight measurements were available for 282 subjects. Subject characteristics at the time of the DXA scans are presented in Table 1, classified by BMD Z-score. Of note, there were differences among the mean age in each group, with a rise in mean age correlating with increasing BMD Z-scores (p <0.01). We did not identify differences between BMD Z-scores and race or ethnicity, with more than 80% of subjects in our sample self-identifying as white (p = 0.40). BMI was significantly different between the groups, with the lowest BMD group having the lowest BMI Z-scores (p < 0.001). Fifteen percent of underweight children and young adults, defined as those with a BMI < 5^th^ percentile for age and gender, had a BMD Z-score ≤ -2, while only 3% of those with BMD Z-score > -1 were underweight (p < 0.01). We also found significant differences in height Z-scores between the groups, with the lowest BMD group having the lowest height Z-scores (p < 0.001). As expected based upon the study design, the mean hip and spine Z-scores were significantly different between the groups (p < 0.001). In our sample population, we also identified that male gender was associated with a BMD Z-score ≤ -2 (p = 0.01).

**Table 1:** Characteristics of study subjects at time of DXA

**Characteristic**		**BMD Z-score ≤ - 2**	**- 2 < BMD Z-score < -1**	**BMD Z-score ≥ -1**	**p**
		**N =102**	**N = 101**	**N = 101**	
**Female**		56 (55%)	63 (62%)	76 (75%)	**0.009**
**Age (years)**	Mean ± SD	13.6 ± 4.1	14.5 ± 4.3	15.5 ± 3.3	**0.003**
**Race/Ethnicity**					**0.397**
	White	88 (86%)	79 (78%)	83 (82%)	
	Black	1 (1%)	2 (2%)	4 (4%)	
	Hispanic	1 (1%)	4 (4%)	3 (3%)	
	Asian	2 (2%)	4 (4%)	3 (3%)	
	Other	6 (6%)	2 (2%)	3 (3%)	
	Not Documented	4 (4%)	10 (10%)	5 (5%)	
**BMI**	Mean ± SD	18.8 ± 3.6	20.2 ± 4.4	23.1 ± 5.2	**<0.001**
	Underweight	13 (15%)	9 (9%)	3 (3%)	**0.003**
	Healthy weight	62 (72%)	75 (76%)	65 (67%)	
	Overweight	9 (11%)	6 (6%)	16 (17%)	
	Obese	2 (2%)	9 (9%)	13 (13%)	
**BMI Z-score**	Mean ± SD	-0.43 + 1.0	-0.11 + 1.2	0.52 + 1.0	**<0.001**
**Height Z-score**	Mean ± SD	-1.2 ± 1.5	-0.7 ± 1.3	-0.1 + 1.1	**<0.001**
**Hip Z-score**	Mean ± SD	-2.18 ± 1.1	-1.0 ± 0.6	0.45 ± 0.8	**<0.001**
**Spine Z-score**	Mean ± SD	-2.4 ± 0.9	-1.1 ± 0.6	0.30 ± 0.9	**<0.001**

Indications for DXA, as documented on the DXA report form from the referring provider, are presented in Table 2. Providers had the option of selecting multiple indications if applicable. We looked at differences in indication by gender, and found that a slightly higher percentage of males were referred for a history of fracture, compared to females (p = 0.05). Additionally, a greater percentage of males reported a history of gastrointestinal disease (p < 0.01), while a greater percentage of females had a history of hypogonadism (p < 0.001). Furthermore, as detailed in the univariate analysis (Table 3), report of a history of a chronic disease associated with bone loss was predictive of a BMD Z-score ≤ -2 (p < 0.001).

**Table 2:** Differences in indication for DXA by gender

**Indication**	**Male**	**Female**	**p**
	**N = 109**	**N= 195**	
**Medical History of Fracture**	47 (43.1%)	62 (31.8%)	**0.05**
History of gastrointestinal disease*	35 (32.1%)	32 (16.4%)	**<0.01**
**Osteopenia noted on a prior x-ray**	9 (8.3%)	21 (10.8%)	**0.48**
**Hypogonadism or delayed puberty**	6 (5.5%)	77 (39.5%)	**<0.001**
**Chronic disease associated with bone loss**	41 (37.6%)	56 (28.7%)	**0.11**

*Includes inflammatory bowel disease, celiac disease, and malabsorption.

**Table 3:** Univariate analysis: Factors associated with BMD Z-score ≤ - 2

**Factor**	**≤ - 2 BMD Z-score**	**BMD Z-score > - 2**	**p**
	**N = 102**	**N= 202**	
**Indication for DXA (reported on referral form)**			
Gastrointestinal disease*	14 (13.7%)	53 (26.2%)	**0.013**
Osteopenia noted on a prior x-ray	13 (12.8%)	17 (8.4%)	**0.232**
Hypogonadism or delayed puberty	23 (22.6%)	60 (29.7%)	**0.186**
Chronic disease associated with bone loss	51 (50.0%)	46 (22.8%)	**<0.001**
**Risk Factors**			
25OHD insufficiency (< 30 ng/mL)	68 (66.7%)	91 (45.1%)	**<0.001**
Family history of osteoporosis	23 (22.6%)	26 (12.9%)	**0.009**
Has reached menarche (females only)	26 (47.3%)	95 (68.4%)	**0.012**
Amenorrhea (females only)	12 (35.3%)	53 (51.5%)	**0.257**
Delayed puberty (males only)	5 (10.9%)	5 (7.8%)	**0.649**
Fracture History**	42 (42.4%)	62 (31.0%)	**0.051**
Eating disorder	8 (7.8%)	26 (12.9%)	**0.189**
Cerebral palsy and/or non-ambulatory	22 (21.6%)	4 (2.0%)	**<0.001**
Cystic fibrosis	6 (5.9%)	7 (3.5%)	**0.371**
Malnutrition §	17 (16.7%)	26 (12.9%)	**0.370**
Malabsorption or IBD	17 (16.7%)	64 (31.7%)	**0.005**
Osteogenisis imperfecta	3 (2.9%)	2 (0.99%)	**0.339**
Recipient of an organ transplant	5 (4.9%)	2 (1.0%)	**0.032**
Prior treatment with radiation and/or chemotherapy	8 (7.8%)	11 (5.5%)	**0.415**
Recipient of a bone marrow transplant	7 (6.9%)	2 (1.0%)	**0.008**
Glucocorticoid use ( > 2 weeks)	39 (38.2%)	74 (36.6%)	**0.785**
Anticonvulsant use	19 (18.6%)	13 (6.4%)	**0.001**
History of prematurity ‡	9 (8.8%)	10 (4.9%)	**0.022**
Height z score (Mean + SD)	-1.15 + 1.5	-0.37 + 1.2	**<0.001**
BMI z score (Mean + SD)	-0.4 + 1.1	0.2 + 1.1	**<0.001**

*Includes inflammatory bowel disease, celiac disease, and malabsorption.

**Excludes patients with Osteogenisis Imperfecta (N=5).

§ Based upon clinical assessment by a nutritionist or physician, as documented in medical record.

‡ Gestational age < 36 weeks.

In the univariate analysis of factors associated with BMD Z-score ≤ -2 (Table 3), we found that correlates of low BMD included low BMI Z-score for age and gender, a history of fracture, a history of 25OHD insufficiency and a family history of osteoporosis. Additionally, we found that female subjects who had not reached menarche at the time of the DXA assessment were at increased risk of low BMD, while amenorrhea was not identified as a risk factor. For young women who had reached menarche, there was no difference in average age of menarche between the two groups (13.1 ± 1.6 years v. 12.8 ± 1.8, p = 0.646).

Medical diagnoses associated with low BMD included cerebral palsy or non-ambulatory state, a history of organ or bone marrow transplant (BMT), treatment with anticonvulsants, and a history of prematurity, defined herein as gestational age < 36 weeks (all at p < 0.05). We did not find a difference (p = 0.27) in duration of anticonvulsant use between subjects with BMD Z-score ≤ -2 (67.9 ± 53.5 months) and those with Z-score > -2 (45.7 ± 45.1 months). Our sample size was too small to analyze the associations between specific anticonvulsants and low BMD. Unexpectedly, both report of a history of a gastrointestinal disease as an indication for DXA (p < 0.01) and history of malabsorption or inflammatory bowel disease (p < 0.01) were associated with a normal BMD.

For all subjects with a fracture history, we did not find a difference (p = 0.63) in mean fracture number between those with BMD Z-score ≤ -2 (3.0 ± 5.0) and those with Z-scores > - 2 (2.7 ± 2.9). We also examined whether the relation between low BMD and fracture history was modified by gender, and found that a history of fracture was associated with an increased risk of a low BMD for males (p < 0.01), but not for females (p = 0.95) (data not shown).

All of the patients with osteogenesis imperfecta (OI) in our cohort were actively treated with bisphosphonates. As a result of bisphosphonate treatment, some subjects with OI had BMD Z-scores that exceeded -2 SD. Additionally, the 5 patients with OI included in this study all had sustained multiple fractures. Since bisphosphonate treatment improves bone density [26-28], we excluded the 5 patients with OI and repeated our univariate analysis to assess the impact of these patients on our results. Without the children with OI, fracture history still remained a risk factor for BMD Z-score ≤ -2 SD only for boys (p = 0.01).

As fracture risk may increase as the BMD Z-score falls below -1 [18], we sought to identify factors associated with this BMD threshold as well. Similar to our initial univariate analysis, BMI < 5^th^ percentile remained a predictor of low BMD. Additionally, a history of cerebral palsy or non-ambulatory state and history of BMT were associated with BMD Z-score < -1.

As there is uncertainty in characterizing (25OHD) values between 20-30 ng/mL, we considered both of these values in assessing for vitamin D insufficiency. For subjects with 25OHD < 30 ng/mL, we did not find a difference (p = 0.24) between mean 25OHD measurements in those with BMD Z-score ≤ -2 (19.3 ± 6.9 ng/mL, n = 67) and those with Z-score > -2 (21.6 ± 14.6 ng/mL, n = 90). 44.8% of subjects with BMD Z-score ≤ -2 and 41.1% of those with Z-score > -2 had a 25OHD measurement < 20 mg/mL, while 66.7% of subjects with BMD Z-score ≤ -2 and 45.1% of those with Z-score > -2 had a 25OHD measurement < 30 mg/mL.

A multivariable logistic regression model was constructed to examine factors associated with a BMD Z-score ≤ -2 (Table 4). Significant predictors include low BMI Z-score, low height Z-score, a 25OHD measurement < 30 ng/mL, and history of a BMT. BMI Z-score was inversely related to the risk of low BMD, with an odds ratio (OR) of 0.52 (95% confidence interval (CI) 0.39 – 0.69). Height Z-score was also inversely related to the risk of low BMD (OR 0.71, CI 0.57, 0.88). We found a notable clinical association between history of 25OHD insufficiency and low BMD, with an almost 4-fold increased risk of low BMD (OR 3.97, CI 2.08, 7.59). In comparison, a history of BMT led to a nearly 6-fold increased risk of low BMD (OR 5.78, CI 1.00, 33.45). Similar to our findings in the univariate analysis, malabsorption or IBD was found to be associated with a normal BMD Z-score in the multivariate analysis. There were no interactions among predictors in the final model. The c-statistic, used to assess the predictive validity of the model, was 0.789.

**Table 4:** Multivariate analysis: Risk factors associated with BMD Z-score ≤ -2

**Factor**	**Odds ratio (95% CI)**	**p**
**BMI z-score**		<0.001
1 unit increase	0.52 (0.39, 0.69)	
**Height z-score**		0.002
1 unit increase	0.71 (0.57, 0.88)	
**Vitamin D Insufficiency**		<0.001
Yes	3.97 (2.08, 7.59)	
Not Documented/No	1.00	
**Malabsorption or IBD**		0.001
Yes	0.29 (0.14, 0.60)	
No	1.00	
**Recipient of a bone marrow transplant**		0.050
Yes	5.78 (1.00, 33.45)	
No	1.00	

BMD Z-score ≤ -2, N = 86; BMD Z-score > -2, N = 196.

c statistic for model = 0.789.

## Discussion

Multiple underlying health problems and associated treatments can impair bone mineral accrual during childhood and adolescence. We sought to identify the risk factors associated with low bone density in subjects referred for DXA measurements of BMD at a tertiary care pediatric hospital to help inform a bone health patient safety initiative. Using a multivariable logistic regression model, we found that low BMI Z-score, history of BMT, and 25OHD insufficiency were the medical conditions most predictive of a BMD Z-score ≤ -2, a threshold deemed by expert consensus to represent a significant low bone density for age [23].

We identified that history of BMT significantly increases the risk of low BMD. Multiple mechanisms have been postulated to mediate the low BMD seen after BMT, including glucocorticoid use, chemotherapy, irradiation, hypogonadism, decreased activity levels, and chronic graft versus host disease [29]. Prior studies support our findings, demonstrating that BMT during childhood increases the risk of low BMD and fracture [29,30], and that bone formation is decreased and bone resorption is increased in the months immediately following BMT [31].

Our multivariate analysis demonstrated that 25OHD insufficiency (< 30 ng/mL) is associated with a nearly 4-fold increased risk of low BMD. This finding is consistent with the observation of Cheng et al. demonstrating that low 25OHD levels correlate with lower cortical BMD in pre-pubertal and pubertal girls [32]. Unlike many of the underlying medical conditions included in our analysis, 25OHD insufficiency may be improved with 25OHD supplementation and thus represents an area amenable to intervention for improved bone health in children and adolescents. Our selection of values less than 30 ng/mL for defining 25OHD deficiency was based upon the lower limit of the normal range of our hospital laboratory. After we designed and initiated our study, the Institute of Medicine reported on dietary reference intakes for calcium and 25OHD, and concluded that an adequate 25OHD level is ≥ 20 ng/mL [33]. However, controversy regarding adequate serum 25OHD levels remains (e.g., above 20 or 30 ng/mL or higher) [34] and additional research is needed to identify the optimal therapeutic target.

We found that low BMI is a significant risk factor for low BMD, yet despite the robust relationship between underweight and low BMD, we did not find a relationship between malnutrition and low bone density. Prior studies have demonstrated a strong relationship between malnutrition in young women with anorexia nervosa, a model of malnutrition-induced bone loss, and low BMD [35-37], which we did not observe in this sample. Bone density in these patients can vary depending on weight loss or gain, and severity of disease. The patients with anorexia nervosa included in this study could have represented a skewed subgroup, with less severe disease and bone loss. A prospective study of larger sample size would likely be powered to reveal the expected relationship between malnutrition and low BMD.

The multivariate analysis demonstrated that low height Z-score is a risk factor for low BMD. Any child with an underlying health problem may have short stature, confounding bone density measurements obtained by DXA [38]. It is important to recognize that DXA measurements may be confounded by bone size, an important issue to consider when studying small underweight adolescents with anorexia nervosa or other pediatric chronic diseases [23]. Zemel et al. recently proposed a method for height adjustment in calculating DXA measurements of bone mass in growing children [39] which will be useful to account for the effect of stature on BMD measurement. Despite these limitations in smaller children, our findings suggest that children who are underweight may be at risk for decreased BMD.

Consistent with prior reports of gender differences in fracture risk [40,41], we found that fractures occurred in a higher percentage of boys than girls in a population referred for DXA measurement. This gender difference has been attributed to higher rates of sports-related injuries in boys [40,41]. Surprisingly, in our univariate analysis of factors associated with BMD Z-score ≤ - 2, we found that male gender was a risk factor for low BMD. This finding is questionable, as there is a known association between male gender and higher BMD [42], and as gender is no longer predictive in the multivariate model, there may be other confounders influencing this finding. As our subjects were selected from a retrospective sample of patients referred for DXA measurements, this observation may also represent selection bias and merits further study.

Prior studies have demonstrated that IBD is both associated with bone loss [43-45], and increased fracture risk in adults [46]. Thus, the finding that a history of malabsorption or IBD was associated with decreased risk of low BMD in the univariate and multivariate analyses was unexpected. The retrospective design of our study may have introduced bias, or referral bias may have influenced the selection of subjects with IBD referred for DXA. Additionally, the timing of the BMD measurements in relation to the IBD diagnosis and associated malabsorption and therapy may have contributed to this finding.

Limitations of this study should be acknowledged. The study design was a retrospective cohort study; therefore, the findings represent associations and causality cannot be inferred. Our results are limited by information obtained solely from subjects referred for DXA measurements at a tertiary care medical center. However, DXA is the assessment tool used most commonly in clinical practice around the world for bone density measurements in children and adults. Thus, we are hopeful that the information gleaned will be useful for clinicians, and in particular, experts in bone health. Measurements of volumetric BMD of the peripheral skeleton were not available and would be valuable to obtain, especially in a cohort of young patients, although this tool is only infrequently used at the present time for clinical purposes. By design, our study was not able to include or identify subjects who were not able to have DXA measurements performed. The retrospective nature of our study required us to rely upon diagnoses and referrals made by providers and the samples sizes in many diagnostic categories were small, limiting our ability to detect differences in BMD. Additionally, due to the reliance on the medical record, we were not able to include assessments of physical activity. We acknowledge that physical activity data would have been useful, as exercise may positively impact BMD [18] and chronic disease often limits activity for a child or adolescent. Obtaining an accurate family history of osteoporosis was also limited by the retrospective nature of this study, as some charts did not include documentation of this information. However, despite these limitations, our results identified the relative risks of a variety of medical conditions known to be associated with low BMD in a sample of patients referred for DXA at a tertiary care center. The information gained from our study highlights pediatric populations who may be at highest risk for a low BMD and may be candidates for bone density screening.

## Conclusions

We used a novel approach to identify factors which lead to the highest risk of low BMD in a pediatric cohort referred for DXA. Prior studies have focused on BMD in specific populations, such as children and young adults with a particular diagnosis or those receiving a certain therapy. Our findings suggest that for all children referred for DXA, a low BMI Z-score, a history of BMT, and vitamin D insufficiency are significant risk factors for a low BMD. Identification of the medical conditions and therapies that lead to the greatest risk of low BMD in a pediatric population is especially important, given that adequate bone mineral accretion during childhood may impact the peak bone mass that is achieved during adulthood, especially for children with chronic medical conditions or therapies. Additionally, we hope that this information can be used to help decrease the frequency of fragility fractures in hospitalized children. Earlier identification of risk factors could potentially lead to the initiation of strategies that maximize bone health during childhood and adolescence and ultimately reduce the future risk for developing osteoporosis.

## Abbreviations

BMD: Bone mineral density; DXA: Dual-energy x-ray absorptiometry; BMI: Body mass index; OR: Odds ratio; CI: Confidence interval; SD: Standard deviation; IBD: Inflammatory bowel disease; BMT: Bone marrow transplant.

## Competing interests

None of the authors have any competing interest relevant to the topic of this manuscript. Catherine Gordon reports that she was the Co-Director of the Clinical Investigator Training Program through Harvard/MIT with support of Pfizer and Merck, through June 2012.

## Authors’ contributions

All authors meet criteria for authorship, have participated in the writing of the manuscript, and have seen and approved the final version. LST and C. Giancaterino wrote the first draft of the manuscript and were responsible for data acquisition. LST and C.Gordon developed the concept and design of the study. PM contributed to study design, writing of the manuscript, and was responsible for statistical analysis. C.Gordon had senior responsibility for overall data interpretation, analysis, and writing. All authors provided critical review and revision for important content. All authors read and approved the final manuscript.

## References

[B1] Stevenson RD Conaway M Chumlea WC Rosenbaum P Fung EB Henderson RC Worley G Liptak G O’Donnell M Samson-Fang L Growth and health in children with moderate-to-severe cerebral palsy Pediatrics 2006 118 3 1010 1018 16950992 10.1542/peds.2006-0298

[B2] Khatri IA Chaudhry US Seikaly MG Browne RH Iannaccone ST Low bone mineral density in spinal muscular atrophy J Clin Neuromuscul Dis 2008 10 1 11 17 18772695 10.1097/CND.0b013e318183e0fa

[B3] Henderson RC Kairalla J Abbas A Stevenson RD Predicting low bone density in children and young adults with quadriplegic cerebral palsy Dev Med Child Neurol 2004 46 6 416 419 15174534 10.1017/s0012162204000672

[B4] Szalay EA Cheema A Children with spina bifida are at risk for Low bone density Clin Orthop Relat Res 2011 469 1253 1257 21042897 10.1007/s11999-010-1634-8 PMC3069300

[B5] Geary DF Schaefer F Comprehensive pediatric nephrology 2008 Philadelphia, PA: Mosby/Elsevier

[B6] Burnham JM Shults J Semeao E Foster B Zemel BS Stallings VA Leonard MB Whole body BMC in pediatric Crohn disease: independent effects of altered growth, maturation, and body composition J Bone Miner Res 2004 19 12 1961 1968 15537438 10.1359/JBMR.040908

[B7] Miller KK Lee EE Lawson EA Misra M Minihan J Grinspoon SK Gleysteen S Mickley D Herzog D Klibanski A Determinants of skeletal loss and recovery in anorexia nervosa J Clin Endocrinol Metab 2006 91 8 2931 2937 16735492 10.1210/jc.2005-2818 PMC3220933

[B8] Turner J Pellerin G Mager D Prevalence of metabolic bone disease in children with celiac disease is independent of symptoms at diagnosis J Pediatr Gastroenterol Nutr 2009 49 5 589 593 19644400 10.1097/MPG.0b013e31819ca18e

[B9] Robertson J Macdonald K Prevalence of bone loss in a population with cystic fibrosis Br J Nurs 2010 19 10 636 639 20622759 10.12968/bjon.2010.19.10.48202

[B10] Caldeira RJ Fonseca Vde M Gomes SC Jr Chaves CR Prevalence of bone mineral disease among adolescents with cystic fibrosis J Pediatr (Rio J) 2008 84 1 18 25 18060251 10.2223/JPED.1737

[B11] Bhudhikanok GS Wang MC Marcus R Harkins A Moss RB Bachrach LK Bone acquisition and loss in children and adults with cystic fibrosis: a longitudinal study J Pediatr 1998 133 1 18 27 9672505 10.1016/s0022-3476(98)70172-6

[B12] Finkelstein JS Klibanski A Neer RM A longitudinal evaluation of bone mineral density in adult men with histories of delayed puberty J Clin Endocrinol Metab 1996 81 3 1152 1155 8772591 10.1210/jcem.81.3.8772591

[B13] Lamberg-Allardt CJ Viljakainen HT 25-Hydroxyvitamin D and functional outcomes in adolescents Am J Clin Nutr 2008 88 2 534S 536S 18689396 10.1093/ajcn/88.2.534S

[B14] Holick MF The role of vitamin D for bone health and fracture prevention Curr Osteoporos Rep 2006 4 3 96 102 16907998 10.1007/s11914-996-0028-z

[B15] Mazziotti G Canalis E Giustina A Drug-induced osteoporosis: mechanisms and clinical implications Am J Med 2010 123 10 877 884 20920685 10.1016/j.amjmed.2010.02.028

[B16] Odame I Duckworth J Talsma D Beaumont L Furlong W Webber C Barr R Osteopenia, physical activity and health-related quality of life in survivors of brain tumors treated in childhood Pediatr Blood Cancer 2006 46 3 357 362 16035080 10.1002/pbc.20512

[B17] Mussa A Bertorello N Porta F Galletto C Nicolosi MG Manicone R Corrias A Fagioli F Prospective bone ultrasound patterns during childhood acute lymphoblastic leukemia treatment Bone 2010 46 4 1016 1020 20044045 10.1016/j.bone.2009.12.019

[B18] Clark EM Ness AR Bishop NJ Tobias JH Association between bone mass and fractures in children: a prospective cohort study J Bone Miner Res 2006 21 9 1489 1495 16939408 10.1359/jbmr.060601 PMC2742714

[B19] Clark EM Tobias JH Ness AR Association between bone density and fractures in children: a systematic review and meta-analysis Pediatrics 2006 117 2 e291 e297 16452336 10.1542/peds.2005-1404 PMC2742730

[B20] Khoury DJ Szalay EA Bone mineral density correlation with fractures in nonambulatory pediatric patients J Pediatr Orthop 2007 27 5 562 566 17585268 10.1097/01.bpb.0000279021.04000.d3

[B21] Skaggs DL Loro ML Pitukcheewanont P Tolo V Gilsanz V Increased body weight and decreased radial cross-sectional dimensions in girls with forearm fractures J Bone Miner Res 2001 16 7 1337 1342 11450710 10.1359/jbmr.2001.16.7.1337

[B22] Ma D Jones G The association between bone mineral density, metacarpal morphometry, and upper limb fractures in children: a population-based case-control study J Clin Endocrinol Metab 2003 88 4 1486 1491 12679427 10.1210/jc.2002-021682

[B23] Gordon CM Bachrach LK Carpenter TO Crabtree N El-Hajj Fuleihan G Kutilek S Lorenc RS Tosi LL Ward KA Ward LM Dual energy X-ray absorptiometry interpretation and reporting in children and adolescents: the 2007 ISCD Pediatric Official Positions J Clin Densitom 2008 11 1 43 58 18442752 10.1016/j.jocd.2007.12.005

[B24] Zemel BS Leonard MB Kalkwarf HJ Specke BL Moyer-Mileur LJ Shepherd JA Cole TJ Pan H Kelly TL Reference data for the whole body, lumbar spine and proximal femur for American children relative to age, gender, and body size Journal of Bone and Mineral Research 2004 19 S231

[B25] Ogden CL Kuczmarski RJ Flegal KM Mei Z Guo S Wei R Grummer-Strawn LM Curtin LR Roche AF Johnson CL Centers for Disease Control and Prevention 2000 growth charts for the United States: improvements to the 1977 National Center for Health Statistics version Pediatrics 2002 109 1 45 60 11773541 10.1542/peds.109.1.45

[B26] Plotkin H Rauch F Bishop NJ Montpetit K Ruck-Gibis J Travers R Glorieux FH Pamidronate treatment of severe osteogenesis imperfecta in children under 3 years of age J Clin Endocrinol Metab 2000 85 5 1846 1850 10843163 10.1210/jcem.85.5.6584

[B27] Munns CF Rauch F Travers R Glorieux FH Effects of intravenous pamidronate treatment in infants with osteogenesis imperfecta: clinical and histomorphometric outcome J Bone Miner Res 2005 20 7 1235 1243 15940378 10.1359/JBMR.050213

[B28] Glorieux FH Bishop NJ Plotkin H Chabot G Lanoue G Travers R Cyclic administration of pamidronate in children with severe osteogenesis imperfecta N Engl J Med 1998 339 14 947 952 9753709 10.1056/NEJM199810013391402

[B29] Taskinen M Saarinen-Pihkala UM Hovi L Vettenranta K Makitie O Bone health in children and adolescents after allogeneic stem cell transplantation: high prevalence of vertebral compression fractures Cancer 2007 110 2 442 451 17549692 10.1002/cncr.22796

[B30] Ruble K Hayat MJ Stewart KJ Chen AR Bone mineral density after bone marrow transplantation in childhood: measurement and associations Biol Blood Marrow Transplant 2010 16 10 1451 1457 20417715 10.1016/j.bbmt.2010.04.010 PMC2933951

[B31] Petryk A Bergemann TL Polga KM Ulrich KJ Raatz SK Brown DM Robison LL Baker KS Prospective study of changes in bone mineral density and turnover in children after hematopoietic cell transplantation J Clin Endocrinol Metab 2006 91 3 899 905 16352681 10.1210/jc.2005-1927

[B32] Cheng S Tylavsky F Kroger H Karkkainen M Lyytikainen A Koistinen A Mahonen A Alen M Halleen J Vaananen K Association of low 25-hydroxyvitamin D concentrations with elevated parathyroid hormone concentrations and low cortical bone density in early pubertal and prepubertal Finnish girls Am J Clin Nutr 2003 78 3 485 492 12936933 10.1093/ajcn/78.3.485

[B33] Institute of Medicine Dietary Reference Intakes for Calcium and Vitamin D 2011 Washington DC: National Academies Press 21796828

[B34] Heaney RP Holick MF Why the IOM recommendations for vitamin D are deficient J Bone Miner Res 2011 26 3 455 457 21337617 10.1002/jbmr.328

[B35] Gordon CM Goodman E Emans SJ Grace E Becker KA Rosen CJ Gundberg CM Leboff MS Physiologic regulators of bone turnover in young women with anorexia nervosa J Pediatr 2002 141 1 64 70 12091853 10.1067/mpd.2002.125003

[B36] Hofman M Landewe-Cleuren S Wojciechowski F Kruseman AN Prevalence and clinical determinants of low bone mineral density in anorexia nervosa Eur J Intern Med 2009 20 1 80 84 19237098 10.1016/j.ejim.2008.04.016

[B37] Misra M Prabhakaran R Miller KK Goldstein MA Mickley D Clauss L Lockhart P Cord J Herzog DB Katzman DK Weight gain and restoration of menses as predictors of bone mineral density change in adolescent girls with anorexia nervosa-1 J Clin Endocrinol Metab 2008 93 4 1231 1237 18089702 10.1210/jc.2007-1434 PMC2291495

[B38] Leonard MB Shults J Elliott DM Stallings VA Zemel BS Interpretation of whole body dual energy X-ray absorptiometry measures in children: comparison with peripheral quantitative computed tomography Bone 2004 34 6 1044 1052 15193552 10.1016/j.bone.2003.12.003

[B39] Zemel BS Leonard MB Kelly A Lappe JM Gilsanz V Oberfield S Mahboubi S Shepherd JA Hangartner TN Frederick MM Height adjustment in assessing dual energy x-ray absorptiometry measurements of bone mass and density in children J Clin Endocrinol Metab 2010 95 3 1265 1273 20103654 10.1210/jc.2009-2057 PMC2841534

[B40] McQuillan R Campbell H Gender differences in adolescent injury characteristics: a population-based study of hospital A&E data Public Health 2006 120 8 732 741 16815504 10.1016/j.puhe.2006.02.011

[B41] Landin LA Epidemiology of children’s fractures J Pediatr Orthop B 1997 6 2 79 83 9165435 10.1097/01202412-199704000-00002

[B42] Kalkwarf HJ Zemel BS Gilsanz V Lappe JM Horlick M Oberfield S Mahboubi S Fan B Frederick MM Winer K The bone mineral density in childhood study: bone mineral content and density according to age, sex, and race J Clin Endocrinol Metab 2007 92 6 2087 2099 17311856 10.1210/jc.2006-2553

[B43] Sylvester FA Wyzga N Hyams JS Davis PM Lerer T Vance K Hawker G Griffiths AM Natural history of bone metabolism and bone mineral density in children with inflammatory bowel disease Inflamm Bowel Dis 2007 13 1 42 50 17206638 10.1002/ibd.20006

[B44] Bianchi ML Inflammatory bowel diseases, celiac disease, and bone Arch Biochem Biophys 503 1 54 65 20599670 10.1016/j.abb.2010.06.026

[B45] Dubner SE Shults J Baldassano RN Zemel BS Thayu M Burnham JM Herskovitz RM Howard KM Leonard MB Longitudinal assessment of bone density and structure in an incident cohort of children with Crohn’s disease Gastroenterology 2009 136 1 123 130 19026647 10.1053/j.gastro.2008.09.072 PMC2705767

[B46] Fickling WE Holdaway A Bhalla AK Robertson DA Prevalence of fragility fracture in inflammatory bowel disease and celiac disease Digestive Disease Week and the 102nd Annual Meeting of the American Gastroenterological Association: 2001 2001 Atlanta, GA: Gastroenterology

